# Assessment of Diversity in the Accessions of *Setaria italica* L. Based on Phytochemical and Morphological Traits and ISSR Markers

**DOI:** 10.3390/molecules24081486

**Published:** 2019-04-15

**Authors:** Bimal Kumar Ghimire, Chang Yeon Yu, Seung-Hyun Kim, Ill-Min Chung

**Affiliations:** 1 Department of Applied Life Science, Konkuk University, Seoul 143-701, Korea; bimal_g12@yahoo.com (B.K.G.); kshkim@konkuk.ac.kr (S.-H.K.); 2 Bioherb Research Institute, Kangwon National University, Chuncheon 200-701, Korea; cyyu@kangwon.ac.kr

**Keywords:** *Setaria italica*, high-performance liquid chromatography, morphological characters, antioxidant activities, antimicrobial activities, accessions

## Abstract

This study was carried out to evaluate genetic diversity, phenolic compound composition, and biological activity of *Setaria italica* L. collected from different parts of South Korea. Antioxidant potential of seeds was estimated by the 1,1-diphenyl-2-picrylhydrazyl (DPPH) radical scavenging assay, and antimicrobial activity was determined by evaluating the minimum inhibitory concentration (MIC). Eight phenolic acids and 3 flavonoids were identified and quantified, among which myricetin and salicylic acid were the most dominant phytochemical compounds detected in the majority of accessions. The antioxidant potential of the leaf extracts of all the accessions was significantly higher (ranging from 32.33 ± 1.53 µg mL^−1^ in SI-03 to 87.87 ± 1.63 µg mL^−1^) in SI-10 than that of the root, stem, or seeds. Among the 15 accessions, methanolic extracts of the SI-15 accession strongly suppressed the growth of *Escherichia coli* (250 µg mL^−1^). Accessions SI-14 and SI-15 showed positive antimicrobial activity against all gram-positive bacteria. Interestingly, extracts of all accessions were more sensitive towards *E. coli* and *Staphylococcus aureus*, with MICs ranging from 250 to 1000 µg mL^−1^. Three phenolic acids, namely chlorogenic acid, catechin, caffeic acid, naringin, hesperetin, and myricetin, were found to be moderately positively correlated with antioxidant activities. A wide range of diversity was observed in morphological traits, namely plant height (99.33 to 201.33 cm), culm length (67.10 to 160.00 cm), spike length (12.80 to 24.00 cm), 1000 seeds weight 1.44 to 2.91 g), bloom beginning (93.67 to 128.00 days), and full bloom (99.67 to 135 days). A dendogram generated from unweighted pair group method with arithmetic mean clustering (UPGMA) cluster analysis based on the morphological traits and inter simple sequence repeat (ISSR) marker data revealed three major groups. However, no clear correlation between these two different approaches was found. The average Shannon’s information index value (I) was 0.492, and it ranged from 0 to 0.693. The average expected heterozygosity (He) was 0.335, and it ranged from 0 to 0.499. The substantial variation in the morphological traits, bioactive properties, and genetic diversity among the accessions may provide useful information for breeding programs attempting to obtain *S. italica* with improved bioactive properties.

## 1. Introduction

*Setaria italica* L. (Foxtail millet), belonging to the Poaceae family, is an important human food in Asia, Europe, and Africa [1]. It is believed to originate from northern China [2,3] and is cultivated worldwide [4,5,6] in acidic and semiarid regions [7]. It has a long history of use, being cultivated since approximately 8700 years ago [1]. Traditionally, the seed flour from these plants was mainly used to prepare pan cakes, porridge, and puddings, among other foods, because of its low allergenic and highly digestible properties [8,9,10,11]. The food products of this plant are safe to use as dietary supplements and are important ingredients in making beverages in Korea, China, and Japan [12]. They are also used as geriatric foods and have diuretic, appetite stimulant, emollient, and digestive properties [10,13]. Consumption of foxtail millet seeds is proposed to be protective against cholera and fever, widely used as an astringent and stomachic, and useful in enhancing virility [14]. The seed extract of this plant is perceived to cure celiac disease [15] and reduce chronic diseases, such as type-2 diabetes [16,17]. The health benefit of this plant has been attributed largely to the presence of various phytochemicals, such as oleic acid, linoleic acid, tocopherol, and phytosterol [18], tannins and phytate [19,20], and alkaloids, phenolics, and flavonoids [21]. In addition, this plant produces a wide range of essential amino acids and minerals [22]. The presence of these chemical compounds also has been attributed to the wide range of biological properties, including antioxidant and hepatoprotective activities [23] as well as antihyperglycemic and hypolipidemic activities [10]. It is believed to lower cholesterol and cancer risk [24], and it possess anti-oxidant, anti-microbial, and anti-carcinogenic properties [21,25,26].

A number of studies reported that phenolic compounds such as phenolic acid and flavonoids not only act as antioxidant compounds [27,28,29] but are also safe for consumption as dietary supplements. A number of previous studies have reported natural antioxidants extracted from plants that effectively inhibited or reduced the formation or scavenging of free radicals and carcinogens [30,31]. Moreover, these phytochemical extracts with potential antimicrobial activities were mainly attributed to the presence of active compounds such as phenols, tannins, alkaloids, terpenoids, and quinones [32,33,34,35,36,37]. *S. italica* has a wide geographical range, which results in variations in nutritional value, biological activity, and agro-morphological traits. Moreover, the antioxidant profiles of *S. italica* accessions that originate from different parts of the Korean peninsula have not been fully examined in earlier studies.

Morphological characterization of collected plant accessions and their documentation can help to determine valuable traits and also important aspects of genetic resources that could serve as initial material for plant breeding programs [38]. Appropriate variables and superior morphological traits from accessions should be carefully and correctly considered for preserving genetic resources, expanding the gene pool, and genetic improvement [39,40]. The identification of important traits that contribute to total diversity and detailed knowledge about the accessions is an important step prior to performing biochemical or molecular studies [41,42]. Moreover, a number of previous studies on ex-situ collections of plants accessions successfully identified the superior accessions with disease resistance [43,44] and root stock production [45,46,47,48]. However, morphological characterization of accession is often considered less reliable for analyzing the genetic diversity of plant species. Various molecular marker have been used to analyse the genetic diversity of plant species including diversity arrays technology (DArT), enotyping by sequencing (GBS), restriction fragment length polymorphism (RFLP), inter simple sequence repeat (ISSR), amplified fragment length polymorphism (AFLP), and simple sequence repeat (SSR) [49,50,51]. The use of ISSR markers has become reliable tools in the field of DNA finger printing and analyzing genetic diversity plant science [52]. Recently, the ISSR marker has been used in diverse plant species due to its quick, robust, efficient, and cost effective process [53]. 

Foxtail millet has a wide geographical range and could vary in its nutritional values, biological activities, and phytochemical composition. To our knowledge, there is no report on the morphological variation and antioxidant and anti-microbial activities of foxtail millet. Moreover, the polyphenol profiles of foxtail millet accessions that originated from different parts of the Korean peninsula have not been fully scrutinized in earlier studies. Therefore, the objectives of the present study were to compare the biological activities in the accessions and their correlations with phenolic compounds. Furthermore, the present comparative study might assist in the selection of elite *S. italica* accessions with superior traits for breeding programs.

## 2. Results and Discussion

### 2.1. Morphological Traits of S. italica Accessions

All 15 accessions of *S. italica* collected from different provinces of South Korea were evaluated for morphological traits (Table 1). There was a wide range of variation in the morphological traits among the accessions (Table 2 and Table 3). Plant height was significantly different (*p* < 0.05) but varied within the studied accessions. Among the different accessions, SI-12 had the greatest plant height (201.33 ± 1.52 cm), whereas SI-04 had the lowest height (99.33 ± 2.52 cm). Plant height was shown to be highly positively correlated with culm length (*r* = 0.800, *p* < 0.01) and also had moderately positive correlations with total average number of leaves (*r* = 0.557, *p* < 0.05), number of nodes (*r* = 0.633, *p* < 0.01), day to bloom beginning (*r* = 0.551, *p* < 0.05), and days to full bloom (*r* = 0.566, *p* < 0.05). Positive correlations have been found between plant height and days to maturity [54]. The greatest leaf length and leaf width were recorded for SI-07 (48.60 ± 1.50 cm and 3.87 ± 0.08 cm, respectively) and lowest values for these parameters were observed in SI-13 (33.23 ± 0.87 cm and 2.13 ± 0.15 cm, respectively), which also had the highest ratio of leaf length to width. The majority of accessions exhibited a single tiller. The accessions had a wide range of variation in culm length, ranging from 67.10 ± 2.01 cm in accession SI-05 to 160.00 ± 2.00 cm in SI-09. Culm length exhibited moderate correlation with the number of nodes (*r* = 0.677, *p* < 0.01), leaf length (*r* = 0.517, *p* < 0.05), number of leaves (*r* = 0.585, *p* < 0.05), days to bloom begin (r = 0.573, *p* < 0.05), and days to full bloom (*r* = 0.607, *p* < 0.05); however, it exhibited negative correlations with 1000 seed weight (*r* = 0.611, *p* < 0.05). A remarkable variation in spike length was observed ranging from 12.80 ± 0.20 cm to 24.00 ± 0.30 cm. A wide range of variation in spike morphological among the accessions was observed (Supplementary Figure S1). The greatest spike length and width were recorded for accession SI-12. Accession SI-04, which had a lower average number of nodes and fewer days to begin blooming and reach full bloom, yielded a higher amount of seed. This study also corroborated the findings of Palakurthi et al. [55]. Interestingly, accession SI-05 had the lowest plant height, spike length, spike width, culm length, and total average number of leaves per plant, and it required fewer days to begin flowering and fewer days to attain full bloom compared to that of the other accessions.

**Figure 1:**
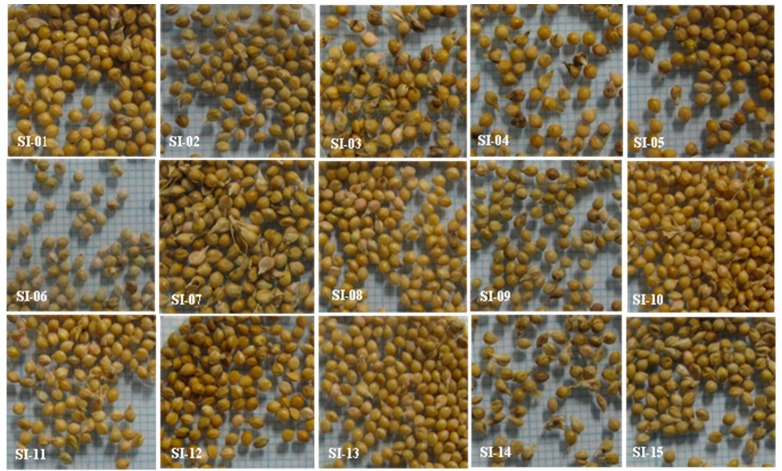
Variation in the seed morphology and color in the 15 accessions of *S. italica*.

In this study, a substantial variation in morphology and weight of 1000 seeds was observed and it ranged from 1.44 ± 0.05 g to 2.91 ± 0.16 g (Figure 1 and Table 3). Our study corroborated the findings of Amgai et al. [54] and Palakurthi et al. [55], wherein they reported phenotypic variation including plant height, 1000 seed weight, mean days to flowering, leaf color, fruit color in *S. italica*. According to them, 1000 seed weight declined when tiller number increased. In a similar study, a positive correlation was observed between grain yield per plants and 1000 grain weight and days to flowering [56,57]. In this study, the total average number of leaves was highly positively correlated with days to bloom begin (*r* = 0.873, *p* < 0.01) and days to full bloom (*r* = 0.874, *p* < 0.01), indicating that leaf number of accessions were a critical factor that directly influenced the initiation of flowering (Table 4). Similarly, days to begin blooming was highly positively correlated with days to full bloom (*r* = 0.998, *p* < 0.01). However, a significant negative correlation existed between number of tillers and number of nodes (*r* = −0.551, *p* < 0.01).

Leaf color and leaf orientation were the only qualitative parameters, and a significant polymorphism was observed within the accessions. Leaf orientation in the majority of accessions was almost horizontal with respect to the stem. Accessions SI-02 and SI-15 were remarkably different from the rest of the accessions, with leaves slightly below the horizontal axis, pointing downwards. It has been argued that leaf orientation is directly associated with plant growth and yield [58,59]. This study corroborated previous findings [54,60,61], wherein a wide variation in grain yield per plant, grain panicle length, plant height, and tillers of *S. italica* plants was found. A number of previous studies attributed the variation in morphological traits to genetic, developmental, and environmental factors [62,63]. In this study, all 15 accessions of *S. italica* were cultivated in the same experimental field with uniform environmental conditions; the differences in the accessions could only be attributed to their origin or their genetic parameters.

### 2.2. Principal Component Analysis 

**Figure 2:**
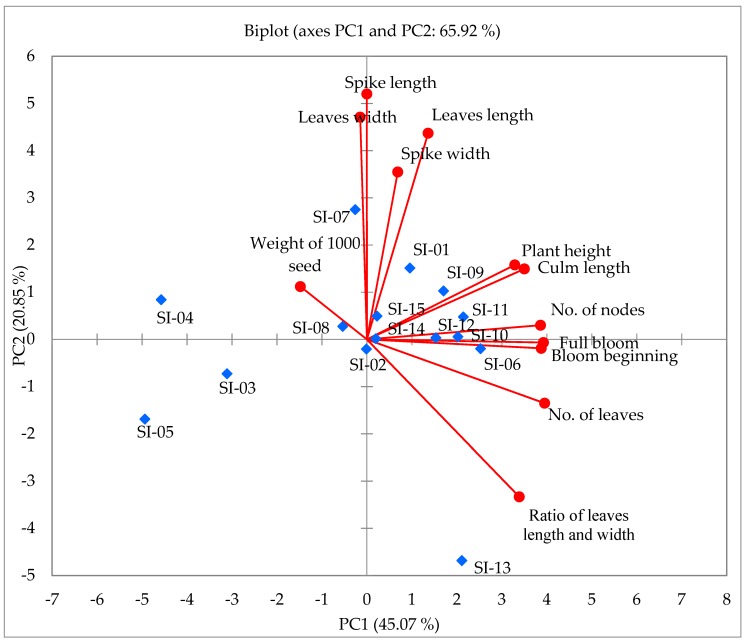
Two-dimensional plot of PC1 and PC2 principal components analysis in the 15 accessions in *S. italica* based on morphological characters.

Principal component analysis (PCA) is a widely used important tool for obtaining overviews of complex datasets. It has also been used for reducing dimensions and revealing relationships among data items [64,65]. Therefore, PCA was applied to assess the variation in the 12 morphological traits in 15 accessions of *S. italica*. The first and second principal components scored 31.440% and 15.206% of the total variance, respectively (Figure 2). As shown in the PCA diagram, we identified different groups of accessions. Along axis 1 of the PCA analysis, four formed a group on the positive side (SI-02, SI-07, SI-09, SI-12) and strongly contributed to morphological traits, such as plant height, spike length, and spike width, whereas the other four accessions (SI-01, SI-06, SI-10, SI-011) formed a group in the negative region of PC2, mainly characterized by morphological traits such as culm length, days to bloom beginning, days to full bloom, number of leaves, number of nodes, leaves length, and ratio (leaf length/leaf width), thus indicating that accessions in this groups are closely related genotypes.

### 2.3. UPGMA Cluster Analysis

**Figure 3:**
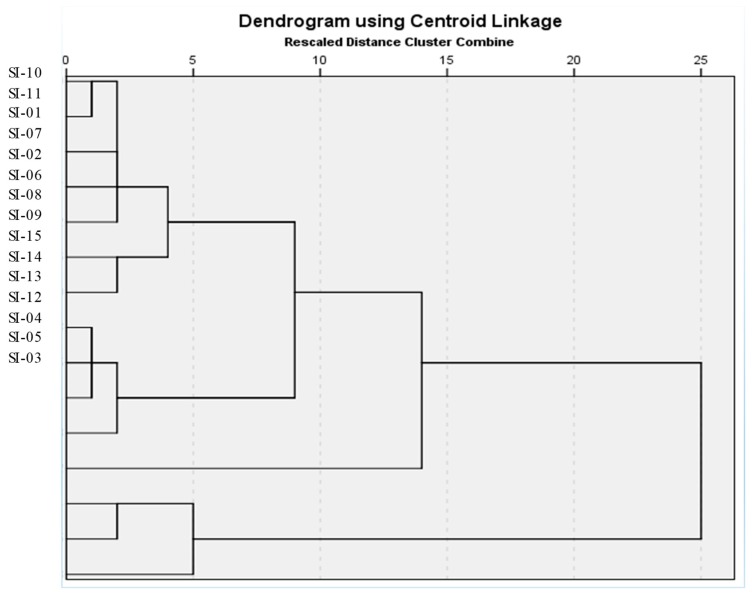
Dendrogram of morphological traits using unweighted pair group method with arithmetic mean clustering (UPGMA) clustering procedures in 15 *S. italica* accessions grown in the field.

The unweighted pair group method with arithmetic mean clustering (UGPMA) cluster analysis of *S. italica* accessions was performed based on the average mean value of morphological traits and could highlight the differences that exist between the accessions. All the studied accessions were grouped into three distinct linkage major branches based on morphological traits (Figure 3). Accession SI-12 formed a separate group, which distinguished it from other groups because of higher plant length and spike length. Accessions SI-03, SI-04, and SI-05 formed a separate group and occupied the extreme end, which possessed high values for similarities in culm and ratio parameters. The remaining accessions formed a single group with two distinct subgroups. The first subgroup comprised accessions SI-08, SI-13, SI-014, and SI-015, whereas the remainder of the accessions formed the second subgroup. However, number of accessions used in this study was relatively small for making accurate generalizations.

### 2.4. Identification and Quantification of Phenolic Compounds of S. italica by HPLC Analysis 

Phenolic compound composition and concentration of 15 accessions of *S. italica* were determined using HPLC. A considerable variation existed in the total phenolic compound contents of Si accessions (Table 5). Among the studied accessions, SI-05 showed the highest value for total phenolic compound concentration (adding all individual compounds) (101.32 ± 1.14 µg mL^−1^ DW), and accession SI-10 exhibited the least amount of total phenolic compounds (8.34 ± 2.00 µg mL^−1^ DW). Fourteen different types of phenolic compounds were identified and quantified, namely *p*-hydroxybenzoic acid, chlorogenic acid, *o*-coumaric acid, ferulic acid, naringin, hesperetin, myricetin, catechin, caffeic acid, syringic acid, salicylic acid, *t*-cinnamic acid, quercetin, and naringenin. Among the phenolic compounds, myricetin (ranging from 1.79 ± 0.26 to 11.19 ± 0.27 µg mL^−1^ DW) was the pre-dominant flavonoid among the accessions. Salicylic acid was the most dominant phenolic acid detected in the majority of the accessions, except for accession SI-14 and SI-15, where it ranged from 0.36 µg mL^−1^ DW to 25.04 ± 1.00 µg mL^−1^ DW. *o*-Coumaric acid was the least abundant phenolic acid, recorded only in accessions SI-10 and SI-11 (0.18 ± 0.18 and 0.45 ± 0.04 µg mL^−1^ DW, respectively). Similarly, naringenin was the least abundant flavonoid, present only in accessions SI-110 and SI-15 (3.64 ± 2.42 and 0.52 ± 0.02 µg mL^−1^ DW, respectively), illustrating the accession-specific characteristics of compounds. The concentration of catechin was the most abundant phenolic compound in accession SI-05, amounting to over 54.62% of the total phenolic compounds in the accession extracts.

### 2.5. In Vitro Antioxidant Capacity of S. italica Accessions

Antioxidant activity of roots, stems, leaves, and seeds of different accessions of *S. italica* determined by DPPH scavenging analysis revealed significant differences (*p* < 0.05) among the studied accessions (Table 6). The antioxidant potential of the leaf extracts for all the accessions were significantly higher (ranging from 32.33 ± 1.53 µg mL^−1^ in SI-03 to 87.87 ± 1.63 µg mL^−1^ in SI-10) than that of the roots, stems, and seeds. Overall antioxidant activity of various accessions revealed that the most inhibiting activity of plant extracts was recorded in leaf extracts of the SI-03 and SI-01 accessions (32.33 ± 1.53 µg mL^−1^ and 32.53 ± 1.20 µg mL^−1^, respectively). However, seed extracts of the cultivated accessions had comparatively lower antioxidant activities. Among the various accession seed samples, the highest DPPH scavenging potential was found in accession SI-09, as indicated by the lower RC50 value 135.17 ± 0.76 µg mL^−1^. The root, leaf, and seed extracts of accession SI-10 were shown to have the least antioxidant activity (176.21 ± 1.06 µg mL^−1^, 87.87 ± 1.63 µg mL^−1^, and 581.33 ± 4.16 µg mL^−1^, respectively) using seed extracts. Variation in the antioxidant activities among the various cultivars of *S. italica* was also observed by Zhang et al. [17] and Kumari et al. [66], and they attributed the variation to the presence of higher concentrations of phenolic compounds. In the present study, a higher concentration of salicylic acid (21.62 ± 1.19 µg mL^−1^) and catechin (7.93 ± 0.11 µg mL^−1^) contents was observed in accession SI-09, which could also have contributed to higher antioxidant activities of the accessions. Moreover, catechin and myricetin were the other phenolic compounds recorded at higher concentrations in the majority of the studied accessions. A significant, moderate correlation was observed between DPPH scavenging activity with chlorogenic acid (*r* = 0.647, *p* < 0.01), catechin (*r* = 0.576, *p* < 0.05), naringin (*r* = 0.681, *p* < 0.01), and hesperetin (*r* = 0.631, *p* < 0.05) (Table 7). A number of studies reported efficient scavenging potential of catechins for DPPH, as well as superoxide and hydroxyl radicals that chelate metal ions or form inactive complexes and inhibit reactive oxygen species (ROS) formation [67,68,69,70,71,72,73,74]. They indicated that catechin possesses the ability for ultra-rapid electron transfer to ROS-induced radical sites on DNA [75] and can neutralize the ROS by forming stable semiquinone free radicals [68]; on the other hand, it may form demonized products, which have been shown to possess higher iron chelating capacity and greater potential to scavenge O^2−^ [76]. Morris and Evans [77] recorded higher antioxidant activities of myricetin. In another study, a strong correlation between the concentration of phytochemicals, such as catechins and myricetin, and its antioxidant properties was found [78,79], indicating that the presence of higher concentrations of these phytochemicals in *S. italica* accessions could possibly contribute to higher antioxidant activities. It is believed that myricetin possesses the ability to protect cells against H_2_O_2_-induced cell disruption by inhibiting ROS generation or preventing H_2_O_2_-induced DNA strand breakage [80]. Moreover, according to Mokrani and Madam [81], the synergism of phenolic compounds presents in plant extracts is likely responsible for higher antioxidant activities. Therefore, various phenolic compounds in the *S. italica* accessions likely produced synergetic effects between phenolic compounds for their antioxidant potential. Further study is essential to determine if these compounds act individually, synergistically, or antagonistically. 

### 2.6. Screening of Antimicrobial Activity of S. italica Accessions Based on MIC

The antimicrobial activity of various accessions of *S. italica* was evaluated using the minimum inhibitory concentration (MIC) method with five different pathogenic bacteria (Table 8). Among the 15 accessions, methanolic extracts of SI-15 accession strongly suppressed the growth of *E. coli* (250 µg mL^−1^). Accessions SI-14 and SI-15 showed positive antimicrobial activity against all the gram-positive bacteria. Interestingly, extracts of all the accessions were more sensitive towards *E. coli* and *S. aureus,* with MICs ranging from 250 to 1000 µg mL^−1^. Microbial strains, such as *S. typhimurium* and *K. pneumonia*, showed the least sensitivity to the majority of the tested accessions. These results are in agreement with the findings of Deepti et al. [82], in which extracts from *Morinda tinctoria* showed strong antimicrobial activities against *E. coli* and *S. aureus* despite the growth of all the accessions in the same environment.

A number of previous studies attributed antimicrobial activity of plant extracts to the phenolic compound composition. In particular, phenolic compounds, such as myricetin and chlorogenic acid, strongly inhibited the growth of *S. pneumoniae* and *E. coli* [83,84]. In other studies, Ikigai et al. [85] and Hermenean et al. [86] recorded complete inhibition of tested microbes (*S. aureus*) by using naringenin. Similarly, other phenolic compounds, such as caffeic acid and catechin, effectively suppressed the growth of *B. subtilis, S. aureus, K. pneumoniae*, and *S. epidermidis* [87]. Therefore, it is likely that the presence of naringenin, chlorogenic acid, and myricetin could possibly contribute to antimicrobial activity of *S. italica* accessions. In this study, the majority of the accessions contained higher concentrations of catechin and myricetin. It has been observed that catechins can bind to the lipid cell membrane of bacteria and causing leakage to the cell membrane [88,89], which results in loss of the ability of the bacteria to bind to the host cells [90] and the inability of the bacteria to produce toxins [91]. Other studies showed that catechins were effectively involved in essential bacterial enzymes, such as cysteine proteinases and protein tyrosine phosphate [92,93], and interfere with DNA replication by reducing the activity of DNA gyrase [94]. Catechins that can easily absorb the alkyl chains of bacterial cell membranes absorb and deteriorate their function [85]. Moreover, some previous studies affirmed that myricetin strongly inhibited the growth of *E. coli* [95]. Furthermore, Cowan et al. [33] demonstrated that higher lipophilic flavonoids could damage the microbial cell membrane and inhibit its growth. Particularly, myricetin can effectively block intracellular ROS [96] and inhibit the growth of gram-positive bacteria more than that of gram-negative bacteria by inhibiting DnaB helicase, an enzyme required in DNA replication and elongation of microbes [97]. Thus, presence of phenolic compounds in the SI accessions could have synergetic or additive effects on antimicrobial activities. Therefore, higher concentrations of catechins in these accessions likely contributed to the antimicrobial activities.

### 2.7. ISSR Marker Polymorphism

The fourteen ISSR primers were designed for observing the DNA banding patterns of collected accessions in *S. italica* (Table 9). In the present study, the percentage of polymorphism ranged from 40–100%, indicating a wide range of diversity within the accessions. A total of 60 polymorphic bands were generated by the fourteen ISSR primers. Obtained bands ranged from 200 bp to 2000 bp (Supplementary Figure S2). A wide range of variation observed in terms of Shannon’s information index (I), number of effective alleles (Ne), number of observed alleles (Na), expected heterozygosity (He), and unbiased expected heterozygosity (uHe) (Table 10). The average Shannon’s information index value (I) was 0.492 ± 0.064 (ranged from 0 to 0.693). The average number of effective alleles (Ne) was 1.59 ± 0.094 (ranged from 1.00 to 1.998), and the average number of observed alleles (Na) was 1.857 ± 0.097 (ranged from 1 to 2). The average expected heterozygosity (He) and unbiased expected heterozygosity (uHe) in the accessions was 0.335 ± 0.047 and 0.34 ± 0.048, respectively. The results indicate that the genetic diversity in the studied accessions was relatively higher and may be attributed to the frequent genetic recombination in the *S. italica*. Dvorakova et al. [98] and Ajitkumar and Pannerselvam [99] reported about the genetic variation between the accessions of *S. italica* using the ISSR marker, which is in accordance with present study. Ardie et al. [100] and Sikdar et al. [101] observed higher polymorphism in the accessions of *S. italica* accessions, although genetic diversity of the accessions was evaluated by using random amplified polymorphic DNA (RAPD) markers. 

**Figure 4:**
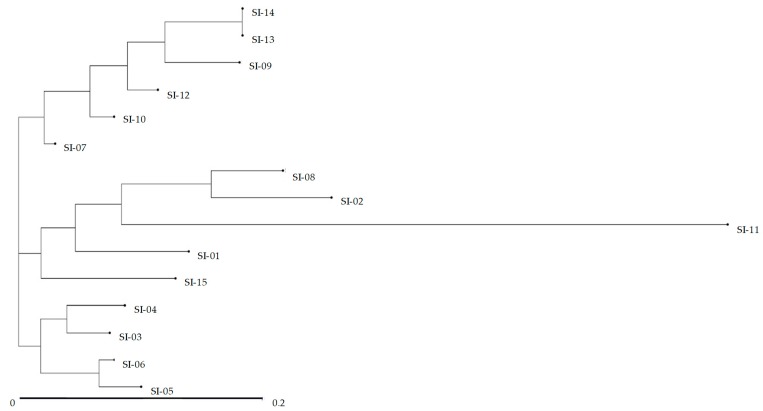
Dendrogram of morphological traits using UPGMA clustering procedures in 15 *S. italica* accessions based on ISSR markers.

**Figure 5:**
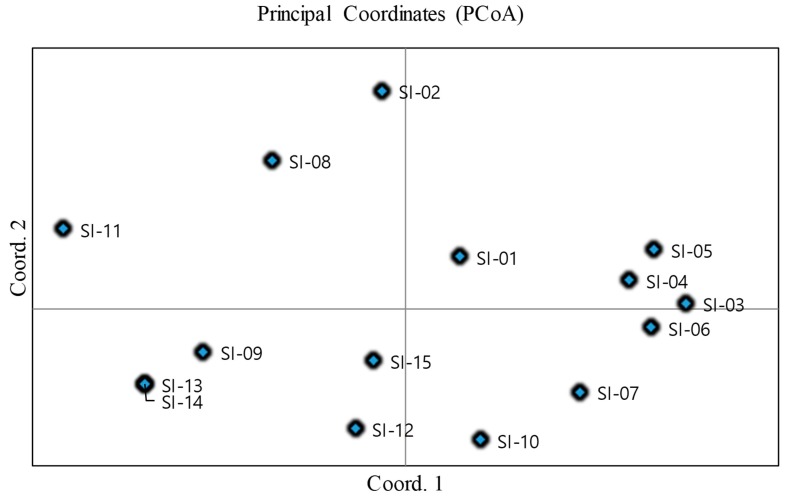
Two-dimensional plot of PC1 and PC2 principal coordinates analysis in the 15 accessions in *S. italica* based on phenolic compounds.

A dendogram generated from UPGMA cluster analysis using ISSR markers revealed three distinct groups (Figure 4). The first group was comprised of six accessions (SI-07, SI-09, SI-10, SI-12, SI-13, and SI-14). The second group consisted of five accessions (SI-01, SI-02, SI-08, SI-11, and SI-15). The third group consisted of rest of the accessions. The clustering pattern clearly indicates a significant level of genetic diversity among the collected accessions. However, the number of group of accessions was the same for morphological and molecular characterization, and significant differences were observed between the clusters analyzed by two different process. A Mantel test between genetic distance and morphological distance did not produce significant correlation (R^2^ = 0.005, *p* < 0.85) (Supplementary Figure S3). The lack of correlation between these two different approaches agreed with the report of Pathak et al. [102] in lychee cultivars, where they observed different identification results using molecular markers and morphological traits. A similar cluster pattern was also reported in the accession of *S. italica* by Dvorakova et al. [98] and Sikdar et al. [101], who used ISSR and RAPD markers, respectively. A principle coordinates analysis (PCoA) was carried out in the 15 accessions of *S. italica* based on ISSR marker to further explore the relationship between the accessions. PC1, PC2, and PC3 accounted for 69.75% of the total variance. However, results obtained from PCoA method found no distinct pattern of clusters (Figure 5).

In summary, the present study was the first report on the antioxidant, antimicrobial, and morphological characterization using *S. italica* accessions. This study further confirms that the ISSR primers are helpful to study genetic diversity of accessions and to identify the closely associated plant species. Based on the morphological traits and ISSR marker data, the 15 accessions of *S. italica* divided into three major groups. However, no clear correlation between these two different approaches was found. The study revealed a significant variation in phenolic compound composition and antioxidant potential of accessions in scavenging DPPH radicals. Among the tested pathogenic microbes, *E. coli* and *S. aureus* proved to be the most sensitive to the seed extract of *S. italica* accessions. No previous study has assessed the wide range of attributes of *S. italica* accessions using an analytical process and presented their chemical composition and its effect on biological activity. However, in the present study, relatively few accessions were tested, and it is recommended to consider more accessions of *S. italica* to elucidate the relationship between biological activity and bioactive compounds in future work. Thus, the present study may act as a morphological marker for future breeding programs to improve yield and functional foods.

## 3. Material and Methods

### 3.1. Chemicals, Standard Compounds, and Solvents

All commercial standard compounds used for analyzing individual phenolic compounds had higher than 99% purity and were purchased from Sigma Aldrich Chemical Co. (St. Louis, MO, USA) and Extrasynthese (Genay Cedex, France). HPLC-grade methanol hexane, ethylacetate, and butanol were supplied by Avantor-J. T. Baker^®^ (Phillipsburg, NJ, USA). Other chemical compounds, such as DPPH, which was used for assessing biological activity, were purchased from Sigma Aldrich Chemical Co. (St. Louis, MO, USA) and Extrasynthese (Genay Cedex, France).

### 3.2. Plant Materials

In this study, we selected 15 superior accessions of *S. italica* collected from different eco-geographical region of Korea were grown during the years 2013, 2014, and 2015 at the Agriculture Research Field, Kangwon National University, South Korea (Figure 1). All the accessions used in this study were grown naturally under ideal and similar plant growth conditions using the same field management.

### 3.3. Identification and Quantification of Phenolic Compound Analysis by HPLC 

The identification and quantification of phenolic compounds from different accessions were carried out using HPLC. Briefly, 1 g of each dried seed sample was crushed to powder and extracted with 10 mL of 80% methanol and shaken in an orbital shaker for 24 h at room temperature (25 °C). To remove debris, all the sample solutions were then filtered through Number 1 Whatman filter paper. Then, the extracted solvent (80% methanol) was evaporated at 40 °C using a vacuum rotary evaporator (Eyela, SB-1300, Shanghai Eyela Co. Ltd., Shanghai, China). The dried solutions were dissolved with 80% methanol (10 mL) to obtain a solution of 50 µg/mL, and then the solution was passed through a 0.45 µm filter unit (TITAN syringe filter nylon membrane) before injection into the HPLC system. The quantification of individual phenolic compounds from each sample was analyzed by HPLC (Shimadzu Instruments CO., LTD, Kyoto, Japan) equipped with a pump (LC-10AD VP) and detector model SPD-M10A, Diode Array detector (280 nm) by following the method described by Thiruvengadam et al. [103] (2016). The chromatographic separation of phenolic compounds was carried out by using an analytical HPLC column (A YMC-Pack ODS-AM-303, 5 μm, 250 × 4.6 mm I.D). The injection volume was 20 µL with the flow rate at 1 mL min^−1^, and the wavelength detected at 280 nm. The mobile phase consisted of solvent A (0.1% glacial acetic acid adjusted in water) and solvent B (0.1% glacial acetic acid in acetonitrile). The gradient elution program was applied as follows: Solvent B was elevated from 8–10% B (2 min); then from 10–30% B (27 min), 30–90% B (50 min), and 90–100% B (52 min), and was then held at 100% of B (57 min). Calibration curves of phenolic compounds were obtained from standards compounds at different concentrations (10, 50, and 100 ppm). Phenolic compounds were identified by matching their retention times with authentic standard phenolic compounds.

### 3.4. Evaluation of Morphological Traits in S. italica Accessions

Seeds of 15 accessions of *S. italica* were kindly provided by the Bio Herb Research Center, Kangwon National University. Cultivation of different accessions of *S. italica* was performed in the experimental farm land of Kangwon National University at Chuncheon, Kangwon-Do, South Korea located at 20°45′S, 42°51′W, with average altitude of 650 m. All the experiments were carried out in a completely randomized block design. There were 10 replicates assigned to each experimental unit. The plots consisted of rows of 70 m in length, spaced 1 m apart with 80 cm between the planted seedlings. The mean minimum and maximum field temperature during the cultivation period were 19.5 °C and 30 °C, respectively, with approximate rainfall rate of 200 nm. The cultivated field was irrigated regularly once weekly by installing a drip-irrigation system until the rainy season started at the beginning of July. The sandy loam texture of the experimental field was maintained at pH of 6.1. The recommended doses of compound fertilizers containing nitrogen, phosphorus, and potassium were applied to the experimental field (N:P:K = 15%:15%:15%) at a rate of 130 kg ha^−1^ during the preparation of field before cultivation of the *S. italica* accessions. Landscape fabric was used as a weed blocker to cover space between the rows. Weeds that emerged within the rows were manually removed regularly during the experimental period. Diseases were controlled by recommended pesticides. Plants were harvested during the first week of October in 2013, 2014, and 2015 when the plants were 110 days old. A total of 15 quantitative and qualitative morphological traits were recorded for each accession. The parameters studied included plant height, culm length, number of leaves, leaf length, leaf color, leaf orientation, leaf width, ratio of leaf length to leaf width tiller number, number of nodes, bloom beginning, full bloom, spike length, spike width, and weight of 1000 seeds. Seed color and seed shape of each accession were considered based on a rating (visual) and were recorded and analyzed. The onset of flower (bloom beginning) was calculated as the number of days between sowing of seeds and the date when the first flower emerged, and full bloom was considered as the number of period (days) between sowing of seeds and when approximately 50% of the flowers had emerged. The mean weight of 1000 seeds was calculated from the weight of 5 randomly selected seed groups from each *S. italica* accession.

### 3.5. Screening of Antioxidant Activity of S. italica Accessions

The antioxidant capacity of the fifteen accessions of *S. italica* was determined and compared by using the 1,1-diphenyl-2-picryl-hydrazyl radical (DPPH) assay by following the method described by Ghimire et al. [104]. Different concentrations of the seed extracts (50 to 1000 ppm) were mixed to 4.5 mL of DPPH (0.004%) in methanol. Then, the mixture was shaken vigorously and allowed to stand for 40 min at 25 °C in the dark condition. The absorbance value of the moisture was recorded using a spectrophotometer (Jasco V530 UV-VIS spectrophotometer, Tokyo, Japan) at 517 nm. Calculated inhibitory concentration (IC_50_) values imply the amount of sample needed to inhibit of 50% DPPH radical present in the mixture. Free radical scavenging activity was calculated by using following equations:

DPPH activity (%) = (A_blank_ − A_sample_)/A_blank_ × 100 (1)

where A_blank_ is the absorbance value of the test reagents without plant extract, and A_sample_ is the absorbance value of the test reagents with plant extract.

### 3.6. Screening of Antimicrobial Activity of S. italica Accessions by Minimum Inhibitory Concentration (MIC)

#### 3.6.1. Microbial Culture

Strains of microorganisms, including *S. aureus* ATCC 13150, *Bacillus subtilis* KCCM 11316, *Salmonella typhimurium* ATCC 14028, *Klebsiella pneumonia* ATCC 9621, and *E. coli* ATCC 43894, were obtained from the Korean Collection Type Culture, Kangwon National University, South Korea. Tested bacterial strains were maintained in a Luria-Bertani (LB) broth containing 5 g L^−1^ bacto-yeast, 10 g L^−1^ bacto-tryptone, 10 g L^−1^ NaCl with a pH of 5.8, and 20% glycerol for 16–24 h at 30 °C. During the MIC assay, the density of bacterial cells in each culture was maintained at 1.5 × 10^8^ CFU mL^−1^. The MIC was defined as the lowest concentration of plant extracts necessary to suppress the growth of pathogenic microorganisms and was estimated by following the process described by Kobayashi et al. [105]. Twofold dilution series of the compounds were prepared in the 96-well assay microplates. Initially, 180 µL of inoculum was transferred into the first row of a 96-well assay microplate (SPC, Life Science Co. Ltd., Seoul, Korea). Then, 20 µL of plant extract solution (1 mg mL^−1^) was added to the first row of the 96-well assay microplates. The final volume in each well was 200 µL. Then, the 96-well plates were incubated for 24 h at 37 °C. Tetracycline (1 mg mL^−1^) was used as a positive control. All measurements of MIC values were performed in triplicate.

#### 3.6.2. DNA Extraction

Young leaves each from fifteen accessions of *S. italica* were collected for DNA extraction. The DNA of individuals accessions were extracted by following the standard CTAB method [106]. Approximately, one gram of fresh leaf from 15 accessions was crushed individually in liquid nitrogen and suspended with extraction buffer (100 mM Tris.HCl pH 8.5, 1.4 mM NaCl, 20 mM EDTA, 2% CTAB, and 0.2% β-mercaptoethanol) and incubated in water bath for 45 min at 65 °C. After incubation, the supernatant was transferred to another 1.5 mL Eppendorf, and an equal volume of ice cold chloroform isoamylalchohol was added. The mixture was then inverted and spun at 11,952× *g* for 10 min. The supernatant was taken into an another eppendorf tube, and an equal volume of ice cold isopropanol was added to the mixture and kept at −20 °C for 40 min. Then, centrifugation was done at 17,226× *g* for 10 min. After discarding the supernatant, 70% ice cold ethanol was added to the DNA pellet. The DNA pellet was air dried and dissolved in 100 μL of nuclease free water. The quality and concentration of DNA genomic DNA of all accessions was determined using UV-VIS spectrophotometer (Jasco V530 UV-VIS spectrophotometer, Tokyo, Japan) and 0.8% agarose gel electrophoresis. Extracted DNA was diluted to 5 ng/µL by using a 1 mMol/L TE buffer.

#### 3.6.3. PCR Amplification and Electrophoresis.

ISSR primers used to amplify the DNA of 15 accessions of *S. italica* were obtained from bioherb research institute, Kangwon National University, South Korea. A total of fifteen ISSR markers with high polymorphism were selected for this study (Table 1). Polymerase chain reaction (PCR) amplification for the genomic DNA of each accession was performed using a 20 µL reaction volume containing 50 ng genomic DNA, 2 µL 1× PCR buffer, and 1 unit tag DNA polymerase—1 µM of primer and 300 µM of dNTPs. PCR conditions for DNA amplification were as follows: An initial denaturation step for 4 min at 95 °C, followed by 45 cycles of denaturation for 30 s at 94 °C, primer annealing for 45 s at 48–52 °C, and extension for 2 min at 72 °C, followed by a final extension for 10 min at 72 °C. The amplified products were loaded onto the 1% gel in 0.5× TBE buffer. The amplified PCR products were electro-phoretically separated on 0.8% agarose in 0.5× TBE buffer for 20 min at 25 V and observed under UV light.

### 3.7. Statistical Analysis

All experiments were repeated at least 3 times. The data shown represent the mean ± SD. The data were statistically evaluated using analysis of variance (ANOVA), and significant differences between the means were assessed using Duncan’s multiple range test at a significance level of *p* < 0.05. Interrelationships among phenotypic traits, phenolic compounds, and antioxidant properties were determined by Pearson’s correlation coefficient using SPSS version 20 (SPSS, IBM, New York, NY, USA, 2011). The principal component analysis (PCA) of morphological traits and phenolic compounds was performed using SPSS version 20. All the quantitative and qualitative morphological traits of accessions were subjected to cluster analysis using the unweighted pair group method with arithmetic averages (UPGMA) using SPSS software version 20. A dendogram was generated using the unweighted pair group method with arithmetic averages (UPGMA) based on ISSR markers. The computation of population genomics like Shannon’s information index (I), number of effective alleles (Ne), number of observed alleles (Na), expected heterozygosity (He), unbiased expected heterozygosity (uHe), and PCoA analysis were were carried out using GenAlEx 6.5 software (Peakall, 2012, Canberra, Australia; Smouse, 2012, New Brunswick, NJ, USA). Similarity coefficients were determined by using the Jaccard index (Jaccard, 1908). A Manital test was performed to assess the correlation between genetic distance and morphological distance using GenAlEx 6.5 software (Peakall and Smouse, 2012).

## Figures and Tables

**Table 1:** Geographical location of accessions used in the study.

Accession Number	Landrace Name	Origin	Province
SI-01	Cheongsilmichajo	Chuncheon-si, sudong-ri	Kangwon-Do, Korea
SI-02	Hwangchajo (Jinju)	Hongcheon-gun, gulji-ri	Kangwon-Do, Korea
SI-03	Mongdangjo	Gapyeong-gun, mokdong-ri	Gyeonggi-Do, Korea
SI-04	Mejo	Wonju-si, hojeo-myeon	Kangwon-Do, Korea
SI-05	Kanghwajo	Yeongwol-gun, macha 9-ri	Kangwon-Do, Korea
SI-06	Neutjo	Yangyang-gun, josan-ri	Kangwon-Do, Korea
SI-07	Kwangjugeurujo	Cheorwon-gun, yukdan 3-ri	Kangwon-Do, Korea
SI-08	Kojangjo	Inje-gun, bupyeong-ri	Kangwon-Do, Korea
SI-09	Eoreunchajo	Taebaek-si, samsu-dong	Kangwon-Do, Korea
SI-10	Saljo	Sokcho-si, joyang-dong	Kangwon-Do, Korea
SI-11	Hwangchajo	Gosung-gun, oho-ri	Kangwon-Do, Korea
SI-12	Sanjeongjo	Pyeongchang-gun, haanmi-ri	Kangwon-Do, Korea
SI-13	Eunchajo	Pocheon-si, udong 2-ri	Gyeonggi-Do, Korea
SI-14	Boksimichajo	Jeongseon-gun, hoedong 5-ri	Kangwon-Do, Korea
SI-15	Bokseulhwangchajo	Gangneung-si, sindang-ri	Kangwon-Do, Korea

**Table 2:** Morphological characteristics of the selected accessions of *S. italica*.

Accessions	Plant Height (cm) **	Culm Length (cm)	Number of Leaves	Leaves Length (cm)	Leaves Width (cm)	Ratio of Leaves Length and Width	Leaves Color	Leaf Orientation	Tiller Number
SI-01	143.17 ± 2.02 ^e^	130.17 ± 1.04 ^k^	11.00 ± 2.00 ^ef^	49.37 ± 0.55 ^h^	2.97 ± 0.16 ^fd^	3.95 ± 0.05 ^d^	Green	2	1
SI-02	160.93 ± 3.00 ^h^	139.66 ± 2.08 ^l^	9.37 ± 0.71 ^cd^	38.50 ± 1.50 ^c^	2.70 ± 0.30 ^cdef^	3.92 ± 0.11 ^d^	Green	3	
SI-03	115.83 ± 2.57 ^c^	98.17 ± 1.04 ^d^	8.33 ± 0.58 ^bc^	40.30 ± 1.13 ^d^	2.33 ± 0.15 ^abc^	3.59 ± 0.09 ^c^	Light green	2	1
SI-04	99.33 ± 2.52 ^b^	80.00 ± 2.00 ^b^	7.17 ± 0.77 ^b^	37.30 ± 1.13 ^bc^	2.67 ± 0.15 ^bcdef^	2.47 ± 0.05 ^a^	Green	2	2
SI-05	84.43 ± 1.50 ^a^	67.10 ± 2.01 ^a^	5.67 ± 0.29 ^a^	36.47 ± 1.50 ^b^	2.49 ± 0.80 ^abcde^	2.30 ± 0.10 ^a^	Green	1	2
SI-06	167.67 ± 1.53 ^i^	150.10 ± 3.00 ^m^	12.17 ± 0.76 ^efg^	46.10 ± 1.02 ^fg^	2.43 ± 0.60 ^abcde^	4.76 ± 0.15 ^f^	Light green	1	1
SI-07	144.10 ± 2.00 ^e^	121.43 ± 2.50 ^i^	9.17 ± 1.04 ^c^	48.60 ± 1.50 ^h^	3.87 ± 0.06 ^h^	2.51 ± 0.08 ^a^	Green	2	1
SI-08	126.60 ± 2.25 ^d^	103.77 ± 1.66 ^e^	10.67 ± 0.28 ^de^	37.17 ± 0.76 ^bc^	2.73 ± 0.25 ^def^	3.33 ± 0.20 ^b^	Green	2	2
SI-09	173.67 ± 1.52 ^j^	160.00 ± 2.00 ^n^	12.23 ± 0.92 ^efg^	46.27 ± 0.75 ^g^	3.20 ± 0.26 ^g^	4.07 ± 0.10 ^de^	Light green	1	2
SI-10	152.50 ± 2.88 ^g^	130.50 ± 1.58 ^k^	12.37 ± 0.75 ^fg^	42.65 ± 0.93 ^e^	2.30 ± 0.24 ^ab^	5.36 ± 0.13 ^g^	Light green	2	1
SI-11	148.00 ± 2.82 ^f^	128.00 ± 1.41 ^j^	13.20 ± 0.98 ^g^	43.50 ± 1.41 ^e^	2.45 ± 0.49 ^abcde^	5.30 ± 0.28 ^g^	Green	2	1
SI-12	201.33 ± 1.52 ^k^	116.16 ± 2.03 ^h^	12.13 ± 0.70 ^efg^	34.30 ± 1.08 ^a^	2.40 ± 0.20 ^abcd^	5.13 ± 0.23 ^g^	Green	2	2
SI-13	127.66 ± 2.51 ^d^	115.50 ± 1.50 ^g^	14.76 ± 0.25 ^h^	33.23 ± 0.87 ^a^	2.13 ± 0.15 ^a^	6.83 ± 0.20 ^h^	Light green	1	1
SI-14	117.13 ± 1.00 ^c^	96.50 ± 0.50 ^c^	12.10 ± 0.36 ^efg^	44.53 ± 0.55 ^efg^	2.80 ± 0.10 ^ef^	4.26 ± 0.15 ^e^	Green	2	1
SI-15	128.20 ± 0.72 ^d^	107.43 ± 0.60 ^f^	12.06 ± 0.30 ^efg^	44.30 ± 0.30 ^ef^	2.92 ± 0.06 ^fg^	4.27 ± 0.15 ^e^	Green	2	1

** The data shown represent the mean ± standard deviation (*n* = 3). Data having the same letter in a row were not significantly differed by Duncan’s multiple range test (*p* < 0.05).

**Table 3:** Morphological characteristics of the selected accessions of *S. italica.*

Accessions	Number of Nodes **	Bloom Beginning (Days)	Full Bloom (Days)	Spike Length (cm)	Spike Width (cm)	Weight of 1000 Seed (g)
SI-01	12.00 ± 2.00 ^cd^	121.00 ± 1.00 ^c^	128.00 ± 2.00 ^c^	22.47 ± 0.50 ^f^	2.90 ± 0.02 ^de^	1.63 ± 0.08 ^b^
SI-02	10.00 ± 2.00 ^bcd^	114.00 ± 2.00 ^b^	121.33 ± 2.52 ^b^	20.43 ± 0.81 ^de^	2.40 ± 0.10 ^b^	1.64 ± 0.07 ^b^
SI-03	7.67 ± 1.53 ^abc^	94.00 ± 2.00 ^a^	99.67 ± 1.53 ^a^	20.37 ± 0.55 ^cde^	2.30 ± 0.10 ^b^	2.17 ± 0.01 ^ef^
SI-04	5.33 ± 1.53 ^a^	93.67 ± 3.51 ^a^	99.67 ± 1.53 ^a^	22.00 ± 1.00 ^f^	3.57 ± 0.12 ^hi^	2.91 ± 0.06 ^i^
SI-05	6.00 ± 1.00 ^ab^	94.33 ± 1.53 ^a^	99.67 ± 1.53 ^a^	17.33 ± 0.58 ^b^	1.93 ± 0.06 ^a^	1.77 ± 0.01 ^bc^
SI-06	14.67 ± 7.37 ^d^	127.67 ± 1.53 ^d^	135.00 ± 2.00 ^d^	19.33 ± 0.76 ^cd^	2.55 ± 0.05 ^bc^	1.83 ± 0.06 ^c^
SI-07	14.00 ± 2.00 ^d^	114.33 ± 2.51 ^b^	121.00 ± 1.00 ^b^	22.00 ± 1.00 ^f^	2.58 ± 0.10 ^bc^	1.98 ± 0.02 ^d^
SI-08	7.67 ± 1.53 ^abc^	128.00 ± 1.00 ^d^	134.67 ± 1.53 ^d^	21.30 ± 0.60 ^ef^	3.27 ± 0.15 ^fg^	2.50 ± 0.10 ^g^
SI-09	13.33 ± 1.52 ^d^	114.00 ± 2.00 ^b^	122.33 ± 1.52 ^b^	19.30 ± 0.20 ^cd^	2.80 ± 0.30 ^cd^	1.44 ± 0.05 ^a^
SI-10	13.25 ± 1.25 ^d^	128.00 ± 1.63 ^d^	135.00 ± 0.82 ^d^	21.62 ± 0.47 ^f^	3.47 ± 0.25 ^gh^	2.13 ± 0.12 ^def^
SI-11	13.25 ± 1.76 ^d^	128.00 ± 2.82 ^d^	135.00 ± 4.24 ^d^	21.95 ± 0.49 ^f^	3.80 ± 0.14 ^i^	2.05 ± 0.07 ^de^
SI-12	13.33 ± 1.04 ^d^	121.67 ± 1.15 ^c^	127.33 ± 3.05 ^c^	24.06 ± 0.30 ^g^	3.16 ± 0.20 ^ef^	2.24 ± 0.21 ^f^
SI-13	14.23 ± 1.56 ^d^	127.66 ± 1.52 ^d^	134.00 ± 1.73 ^d^	12.80 ± 0.20 ^a^	1.70 ± 0.20 ^a^	1.71 ± 0.07 ^bc^
SI-14	14.06 ± 0.60 ^d^	121.30 ± 0.60 ^c^	128.16 ± 0.76 ^c^	19.26 ± 0.64 ^c^	2.81 ± 0.07 ^cd^	2.75 ± 0.05 ^h^
SI-15	11.96 ± 0.45 ^cd^	122.33 ± 3.21 ^c^	128.00 ± 0.90 ^c^	22.10 ± 0.52 ^f^	2.36 ± 0.15 ^b^	2.62 ± 0.06 ^gh^

** The data shown represent the mean ± standard deviation (*n* = 3). Data having the same letter in a row were not significantly differed by Duncan’s multiple range test (*p* < 0.05).

**Table 4:** Pearson’s correlation coefficients between the main morphological characteristics in *S. italica* accessions.

Analytes	Plant Height	Culm Length	Number of Leaves	Leaves Length	Leaves Width	Ratio of Leaf L/W	Number of Nodes	Bloom Beginning	Full Bloom	Spike Length	Spike Width	Weight of 1000 Seed	Tiller Number
Plant height	1	0.800 **	0.557 *	0.187	0.072	0.471	0.653 **	0.551 *	0.566 *	0.348	0.478	−0.379	−0.019
Culm length		1	0.585 *	0.517 *	0.198	0.442	0.677 **	0.573 *	0.607 *	0.108	0.096	−0.611 *	−0.298
Number of leaves			1	0.135	−0.195	0.893 **	0.823 **	0.873 **	0.874 **	−0.156	0.168	−0.129	−0.421
Leaves length				1	0.636 *	−0.162	0.447	0.205	0.232	0.333	−0.062	−0.173	−0.502
Leaves width					1	−0.548 *	0.147	−0.067	−0.046	0.332	−0.070	−0.050	0.010
Ratio of leaf L/W						1	0.675 **	0.707 **	0.700 **	−0.318	0.094	−0.213	−0.418
Number of nodes							1	0.754 **	0.763 **	−0.067	0.077	−0.294	−0.551 *
Bloom beginning								1	0.998 **	0.020	0.228	−0.100	−0.381
Full bloom									1	0.017	0.224	−0.125	−0.378
Spike length										1	0.732 **	0.365	0.105
Spike width											1	0.366	0.329
Weight of 1000 seed												1	0.085
Tiller number													1

** Correlation is significant at the 0.01 level (2-tailed). * Correlation is significant at the 0.05 level (2-tailed).

**Table 5:** Distribution of total phenolic compounds in the different accession of *S. italica*.

**Accessions**	*p*-HY *	**CH**	**CAT**	**CA**	**SY**	**SA**	***o*-C**	
	--------------------------------------------------------------------------------------(µg/mL) **-------------------------------------------------							
SI-01	0.96 ± 0.06 ^c^	0 ^a^	31.41 ± 0.52 ^d^	0.41 ± 0.03 ^a^	8.63 ± 0.40 ^d^	9.92 ± 0.39 ^h^	0 ^a^	
SI-02	0.41 ± 0.02 6^b^	0 ^a^	0 ^a^	0 ^a^	0 ^a^	0.36 ± 0.04 ^ab^	0 ^a^	
SI-03	0 ^a^	1.22 ± 2.00 ^bc^	0 ^a^	0 ^a^	0.16 ± 0.02 ^a^	0.84 ± 0.06 ^abc^	0 ^a^	
SI-04	0 ^a^	0.35 ± 0.05 ^b^	21.27 ± 0.05 ^c^	1.50 ± 0.05 ^a^	7.32 ± 0.20 ^b^	5.26 ± 0.05 ^e^	0 ^a^	
SI-05	1.40 ± 0.56 ^d^	0 ^a^	55.34 ± 0.58 ^e^	0.56 ± 0.05 ^a^	17.97 ± 0.06 ^f^	23.06 ± 1.01 ^k^	0 ^a^	
SI-06	0 ^a^	0.35 ± 0.05 ^b^	34.53 ± 0.50 ^d^	0.43 ± 0.02 ^a^	7.79 ± 0.17 ^c^	9.37 ± 0.55 ^h^	0 ^a^	
SI-07	0 ^a^	0 ^a^	0 ^a^	0 ^a^	0 ^a^	1.36 ± 0.15 ^bc^	0 ^a^	
SI-08	0 ^a^	0 ^a^	11.62 ± 0.54 ^b^	6.83 ± 1.42 ^b^	0 ^a^	25.04 ± 1.00 ^l^	0 ^a^	
SI-09	0 ^a^	9.28 ± 0.03 ^e^	67.93 ± 0.11 ^f^	12.25 ± 0.03 ^c^	0 ^a^	21.62 ± 1.19 ^j^	0 ^a^	
SI-10	0 ^a^	0.59 ± 1.19 ^ab^	0 ^a^	0 ^a^	0 ^a^	1.69 ± 0.96 ^c^	0.18 ± 0.18 ^b^	
SI-11	0 ^a^	2.45 ± 0.35 ^d^	0 ^a^	0 ^a^	0 ^a^	3.15 ± 0.05 ^d^	0.45 ± 0.04 ^c^	
SI-12	0 ^a^	0.69 ± 0.04 ^ab^	20.25 ± 9.00 ^c^	0.29 ± 0.01 ^a^	7.36 ± 0.06 ^b^	7.15 ± 0.06 ^f^	0 ^a^	
SI-13	0.44 ± 0.05 ^b^	0.28 ± 0.03 ^a^	35.21 ± 0.26 ^d^	0.55 ± 0.05 ^a^	10.48 ± 0.50 ^e^	8.19 ± 0.08 ^g^	0 ^a^	
SI-14	0 ^a^	1.39 ± 0.13 ^c^	0 ^a^	0 ^a^	0 ^a^	0 ^a^	0 ^a^	
SI-15	0 ^a^	0 ^a^	0 ^a^	0 ^a^	0 ^a^	0 ^a^	0 ^a^	
**Accessions**	**FE**	**NA**	**HN**	**MY**	***t*-C**	**QU**	**NE**	**Total Phenolic Compound**
	--------------------------------------------------------------------------------------(µg/mL) *-----------------------------------------------------------							
SI-01	0 ^a^	0 ^a^	0 ^a^	0^a^	0 ^a^	0 ^a^	0 ^a^	51.23 ± 1.08 ^a^
SI-02	8.28 ± 0.20 ^bc^	0 ^a^	0 ^a^	1.79 ± 0.26 ^b^	0.05 ± 0.01 ^c^	0 ^a^	0 ^a^	10.63 ± 0.55 ^a^
SI-03	3.27 ± 0.15 ^ab^	0.61 ± 0.10 ^cd^	0.76 ± 0.05 ^d^	2.30 ± 0.20 ^b^	0 ^a^	10.99 ± 0.08 ^d^	0 ^a^	20.09 ± 1.01 ^a^
SI-04	0 ^a^	0.28 ± 0.03 ^ab^	0 ^a^	7.39 ± 0.17 ^h^	0 ^a^	0 ^a^	0 ^a^	44.37 ± 0.55 ^a^
SI-05	0 ^a^	0.40 ± 0.05 ^bc^	0.37 ± 0.03 ^c^	3.06 ± 0.05 ^c^	0 ^a^	0 ^a^	0 ^a^	101.32 ± 1.14 ^ab^
SI-06	2.10 ± 2.77 ^a^	0 ^a^	0.66 ± 0.05 ^d^	3.82 ± 0.11 ^d^	0 ^a^	0 ^a^	0 ^a^	57.14 ± 1.03 ^a^
SI-07	1.98 ± 0.06 ^a^	0 ^a^	0 ^a^	2.21 ± 0.25 ^b^	0 ^a^	0.66 ± 0.05 ^c^	0 ^a^	6.21 ± 0.80 ^a^
SI-08	0 ^a^	0 ^a^	0.91 ± 0.03 ^e^	5.61 ± 0.60 ^g^	0 ^a^	0 ^a^	0 ^a^	43.25 ± 1.09 ^a^
SI-09	0 ^a^	9.32 ± 0.08 ^g^	3.77 ± 0.20 ^f^	23.96 ± 0.45 ^j^	0 ^a^	0 ^a^	0 ^a^	152.83 ± 1.06 ^b^
SI-10	4.47 ± 8.94 ^ab^	0.25 ± 0.49 ^ab^	0.05 ± 0.10 ^a^	2.41 ± 0.89 ^bc^	0.02 ± 0.03 ^ab^	0.42 ± 0.28 ^b^	3.64 ± 2.42 ^b^	13.74 ± 10.06 ^a^
SI-11	17.50 ± 0.71 ^d^	1.06 ± 0.08 ^e^	0.19 ± 0.01 ^b^	3.75 ± 0.35 ^d^	0.04 ± 0.01 ^bc^	0 ^a^	0 ^a^	28.50 ± 0.71 ^a^
SI-12	0 ^a^	0 ^a^	0 ^a^	4.61 ± 0.34 ^ef^	0 ^a^	0 ^a^	0 ^a^	39.88 ± 0.83 ^a^
SI-13	0 ^a^	0 ^a^	0 ^a^	8.70 ± 0.26 ^h^	0 ^a^	0 ^a^	0 ^a^	62.88 ± 2.59 ^ab^
SI-14	11.43 ± 0.51 ^c^	0.75 ± 0.10 ^d^	0 ^a^	5.22 ± 0.25 ^fg^	0.72 ± 0.03 ^e^	0 ^a^	0 ^a^	20.12 ± 2.80 ^a^
SI-15	20.52 ± 0.50 ^d^	1.69 ± 0.28 ^f^	0.25 ± 0.05 ^bc^	11.19 ± 0.27 ^i^	0.36 ± 0.03 ^d^	0 ^a^	0.52 ± 0.02 ^a^	34.48 ± 0.50 ^a^

* Abbreviation: *p*-HY: *p*-Hydroxybenzoic acid, CH: Chlorogenic acid, CAT: Catechin, CA: Caffeic acid, SY: Syringic acid, SA: Salicylic acid, *o*-C: *o*-Coumaric acid, FE: Ferulic acid, NA: Naringin, HN: Hesperetin, MY: Myricetin, *t*-C: *t*-Cinnamic acid, QU: Quercetin, NE: Naringenin. ** The values of individual compounds are the mean ± standard deviation (*n* = 3). Data having the same letter in a row were not significantly differed by Duncan’s multiple range test (*p* < 0.05).

**Table 6:** Antioxidant activity of the selected accessions of *S. italica.*

Accessions	Root	Stem	Leaf	Seed
	RC_50_ (µg mL^−1^) *			
SI-01	93.27 ± 0.25 ^g^	138.00 ± 1.00 ^f^	32.53 ± 1.20 ^a^	159.67 ± 1.50 ^b^
SI-02	140.33 ± 0.58 ^j^	222.43 ± 2.14 ^k^	60.20 ± 1.05 ^e^	310.00 ± 1.00 ^i^
SI-03	78.53 ± 0.50 ^d^	99.43 ± 0.51 ^a^	32.33 ± 1.53 ^a^	252.00 ± 2.00 ^d^
SI-04	100.00 ± 1.00 ^h^	198.67 ± 1.16 ^i^	39.10 ± 1.02 ^b^	270.00 ± 2.00 ^f^
SI-05	71.10 ± 1.02 ^c^	118.00 ± 1.00 ^c^	60.00 ± 1.00 ^e^	280.00 ± 2.00 ^g^
SI-06	58.20 ± 1.06 ^ab^	198.33 ± 1.53 ^i^	59.10 ± 1.02 ^e^	314.67 ± 4.16 ^j^
SI-07	118.53 ± 1.50 ^i^	142.20 ± 2.03 ^g^	37.10 ± 1.02 ^b^	210.33 ± 1.53 ^c^
SI-08	56.20 ± 1.06 ^a^	120.77 ± 1.57 ^d^	72.17 ± 1.26 ^f^	309.83 ± 1.23 ^i^
SI-09	88.00 ± 1.00 ^f^	107.67 ± 1.53 ^b^	47.90 ± 1.16 ^c^	135.17 ± 0.76 ^a^
SI-10	176.21 ± 1.06 ^k^	132.67 ± 1.53 ^e^	87.87 ± 1.63 ^g^	581.33 ± 4.16 ^k^
SI-11	58.17 ± 1.26 ^ab^	118.33 ± 1.53 ^cd^	48.77 ± 1.57 ^c^	270.00 ± 2.00 ^f^
SI-12	79.83 ± 1.76 ^d^	119.67 ± 1.53 ^cd^	51.20 ± 1.06 ^d^	280.67 ± 3.05 ^g^
SI-13	60.07 ± 1.40 ^b^	239.10 ± 1.02 ^l^	48.00 ± 1.00 ^c^	259.33 ± 5.03 ^e^
SI-14	83.00 ± 3.00 ^e^	177.87 ± 0.23 ^h^	50.83 ± 0.76 ^d^	286.33 ± 2.52 ^h^
SI-15	100.33 ± 1.53 ^h^	298.53 ± 1.50 ^m^	48.00 ± 2.00 ^c^	270.00 ± 2.00 ^f^

* The values of individual compounds are the mean ± standard deviation (*n* = 3). Data having the same letter in a row were not significantly differed by Duncan’s multiple range test (*p* < 0.05).

**Table 7:** Pearson’s correlation coefficients between the antioxidant potential and phenolic compounds in *S. italica* accessions.

Analytes	Phenolic Compound													
	*p*-HY ^1^	CH	CAT	CA	SY	SA	*o*-C	FE	NA	HN	MY	*t*-C	QU	NE
DPPH	0.166	0.647 **	0.557 *	0.576 *	0.011	0.332	−0.225	−0.243	0.681 **	0.631 *	0.568 *	−0.137	−0.005	−0.468

** Correlation is significant at the 0.01 level (2-tailed). * Correlation is significant at the 0.05 level (2-tailed). ^1^ Abbreviation: *p*-HY: *p*-Hydroxybenzoic acid, CH: Chlorogenic acid, CAT: Catechin, CA: Caffeic acid, Syringic acid, SA: Salicylic acid, *o*-C: *o*-Coumaric acid, FE: Ferulic acid, NA: Naringin, HN: Hesperetin, MY: Myricetin, *t*-C: *t*-Cinnamic acid, QU: Quercetin, NE: Naringenin.

**Table 8:** Antimicrobial activities of the selected accessions of *S. italica*.

Accessions	MIC (μg/mL) *				
	*S. aureus*	*B. subtilis*	*S. typhimurium*	*K. pneumonia*	*E. coli*
SI-01	1000	1000	>1000	>1000	500
SI-02	1000	1000	>1000	>1000	500
SI-03	1000	>1000	>1000	>1000	1000
SI-04	1000	>1000	>1000	>1000	500
SI-05	1000	1000	>1000	>1000	500
SI-06	1000	1000	>1000	>1000	500
SI-07	1000	1000	>1000	1000	500
SI-08	1000	1000	500	>1000	500
SI-09	1000	1000	>1000	>1000	500
SI-10	1000	>1000	>1000	>1000	500
SI-11	1000	1000	>1000	>1000	500
SI-12	1000	1000	>1000	>1000	500
SI-13	1000	1000	>1000	>1000	500
SI-14	1000	1000	500	500	500
SI-15	1000	1000	1000	500	250

* The MIC values against bacteria were determined by the serial 2-fold dilution method. The growth of the bacteria was evaluated by the degree of turbidity of the culture measured with the naked eye.

**Table 9:** Inter simple sequence repeat (ISSR) marker used for genetic diversity of *S. italica*.

ISSR Primer	Base Sequence (5′-3′)	Total Number of Bands	Number of Polymorphic Bands	Percentage of Polymorphism (%)
ISSR1	(CTG)^7^G	5	5	100
ISSR2	(GAG)^6^C	2	2	100
ISSR3	(GAC)^6^T	3	3	100
ISSR4	(GACA)^5^	6	4	66.67
ISSR5	(GTC)^6^A	4	3	75.00
ISSR6	(GTG)^6^C	3	3	100
ISSR7	(CTC)^7^	7	7	100
ISSR8	(GTG)^6^A	6	5	83.33
ISSR9	A(CACA)^3^CACTG	6	4	100
ISSR10	(GAC)^6^	5	5	100.00
ISSR11	(GACA)^4^	8	3	76.67
ISSR12	(CTC)^7^A	6	5	70.00
ISSR13	(GACA)^4^A	7	4	40.00
ISSR14	(TC)^8^AG	6	7	75.70
Total		74	60	

**Table 10:** Genetic diversity of 15 accessions of *S. italica* assessed by ISSR markers.

Primer	P *	q	Na	Ne	I	He	uHe
ISSR1	0.106	0.894	2.000	1.233	0.337	0.189	0.195
ISSR 2	0.484	0.516	2.000	1.998	0.693	0.499	0.517
ISSR 3	0.553	0.447	2.000	1.978	0.688	0.494	0.511
ISSR 4	1.000	0.000	1.000	1.000	0.000	0.000	0.000
ISSR 5	1.000	0.000	1.000	1.000	0.000	0.000	0.000
ISSR 6	0.368	0.632	2.000	1.869	0.658	0.465	0.481
ISSR 7	0.184	0.816	2.000	1.428	0.477	0.300	0.310
ISSR 8	0.368	0.632	2.000	1.869	0.658	0.465	0.481
ISSR 9	0.742	0.258	2.000	1.621	0.571	0.383	0.396
ISSR 10	0.742	0.258	2.000	1.621	0.571	0.383	0.396
ISSR 11	0.106	0.894	2.000	1.233	0.337	0.189	0.195
ISSR 12	0.368	0.632	2.000	1.869	0.658	0.465	0.481
ISSR 13	0.368	0.632	2.000	1.869	0.658	0.465	0.481
ISSR 14	0.270	0.730	2.000	1.650	0.583	0.394	0.408
Mean	0.475	0.524	1.857	1.588	0.492	0.335	0.347
SE	0.286	0.286	0.097	0.094	0.064	0.047	0.048

* p & q, estimated Allele Frequency; Na, observed number of alleles; Ne, effective number of alleles; I, Shannon’s information index; expected heterozygosity (He), unbiased expected heterozygosity (uHe).

## Supplementary Materials

The following are available online at https://www.mdpi.com/1420-3049/24/8/1486/s1, Figure S1. Variation in the spike morphology in the 15 accessions of *S. italica*. Figure S2. Banding pattern generated by ISSR-9 primer. M: DNA ladder, 1-15 represent the DNA banding pattern of fifteen accessions of *S. italica.* Figure S3. Correlation (R^2^) between genetic and morphological distances in fifteen accessions of *S. italica* based on the Mantel test.
