# Supplementary material for: Assessment of Diversity in the Accessions of Setaria italica L. Based on Phytochemical and Morphological Traits and ISSR Markers
Source: Molecules. 2019 Apr 15;24(8):1486. doi: 10.3390/molecules24081486 (PMC6514597; doi:10.3390/molecules24081486)
Supplement: Supplementary file 1 [file molecules-24-01486-s001.pdf]

Assessment of Diversity in the Accessions of *Setaria italica* L. Based on Phytochemical and Morphological Traits and ISSR Markers

Bimal Kumar Ghimire [1], Chang Yeon Yu [2], Seung-Hyun Kim [1] and Ill-Min Chung [1,*]

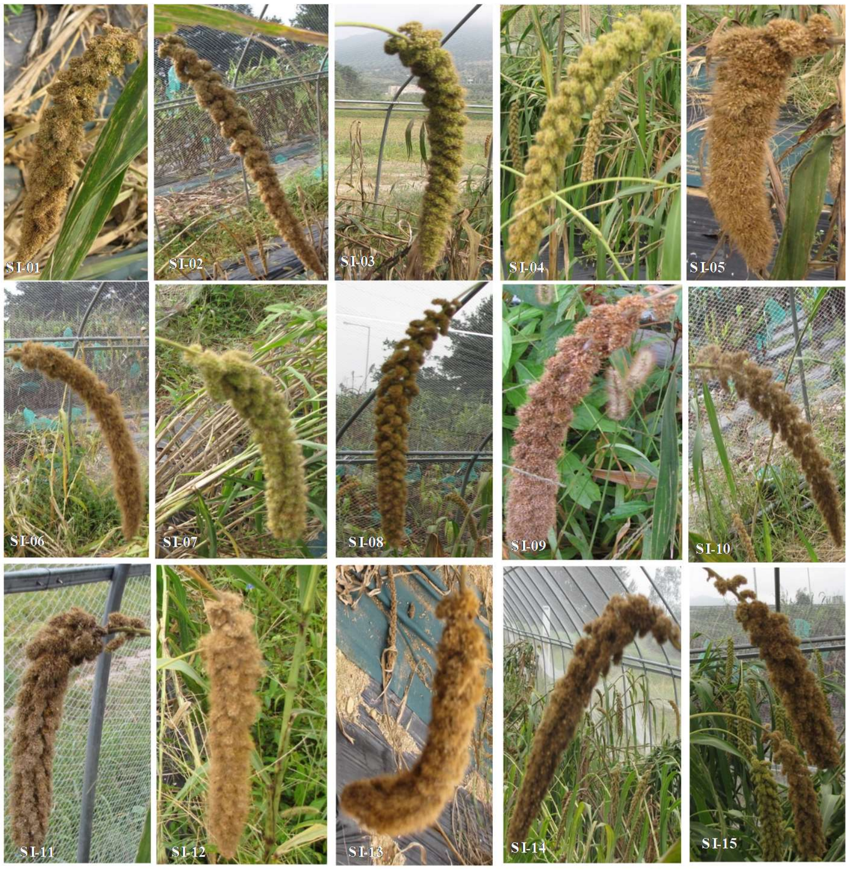

Figure 1. Variation in the spike morphology in the 15 accessions of *S. italica*.

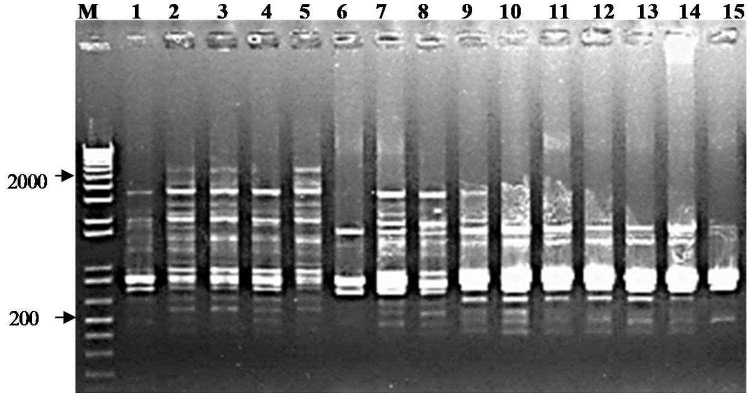

Figure 2. Banding pattern generated by ISSR-9 primer. M: DNA ladder, 1-15 represent the DNA banding pattern of fifteen accessions of *S. italica.*

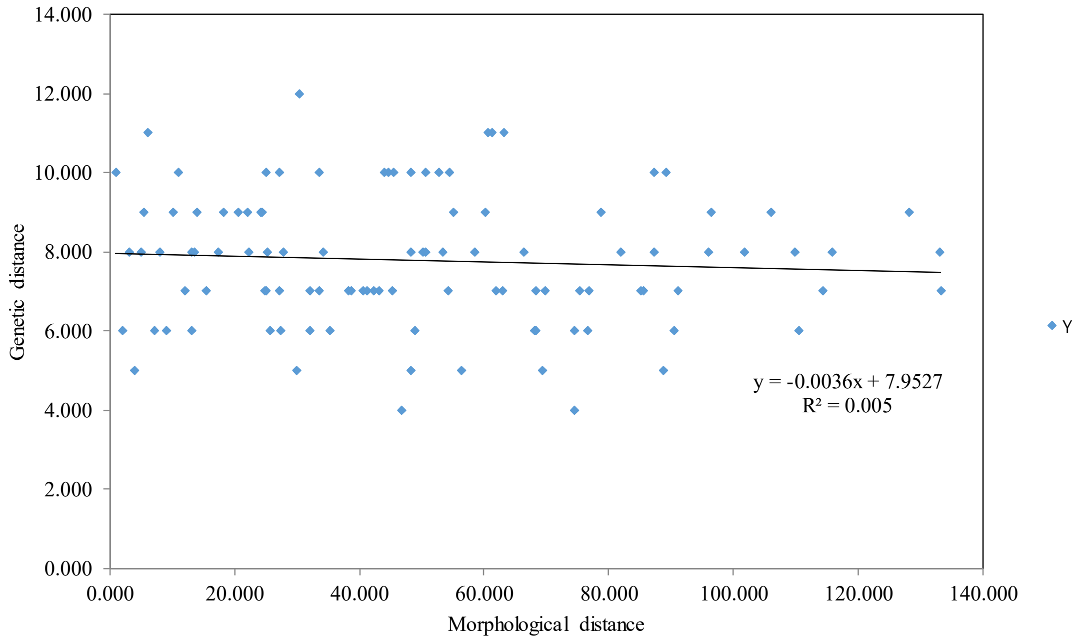

Figure 3. Correlation (R^2) between genetic and morphological distances in fifteen accessions of *S. italica* based on the Mantel test.
